# Nonaaqua­praseodymium triiodide–thio­urea (1/2)

**DOI:** 10.1107/S1600536811054663

**Published:** 2012-01-07

**Authors:** Taisia A. Antonenko, Lyudmila Yu. Alikberova, Dmitry V. Albov

**Affiliations:** a Department of Inorganic Chemistry, M. V. Lomonosov Moscow State University of Fine Chemical Technologies, 86 Vernadskogo Av., Moscow 119571, Russian Federation; b Chemistry Department, Moscow State University, Leninskiye Gory, Moscow 119992, Russian Federation

## Abstract

The title compound, [Pr(H_2_O)_9_]I_3_·2CS(NH_2_)_2_, an adduct of nona­aqua­praseodymium triiodide with two thio­urea mol­ecules, is composed from [Pr(H_2_O)_9_]^3+^ cations (polyhedron: monocapped tetra­gonal anti­prism), noncoordinated thio­urea mol­ecules and iodide anions. The components are evidently connected by hydrogen bonds but in the presence of heavy atoms water H atoms have not been located. The complex cation and one of the two independent iodide anions are located on a twofold axis.

## Related literature

For related compounds, see: Romanenko *et al.* (1980 ▶, 1981**a* ▶,b* ▶, 1985 ▶,1986 ▶); Antonenko *et al.* (2011 ▶). For applications of similar complexes, see: Suponitsky *et al.* (1988 ▶). For titration methods, see: Patrovsky (1959 ▶); Kolthoff & Belcher (1957 ▶).
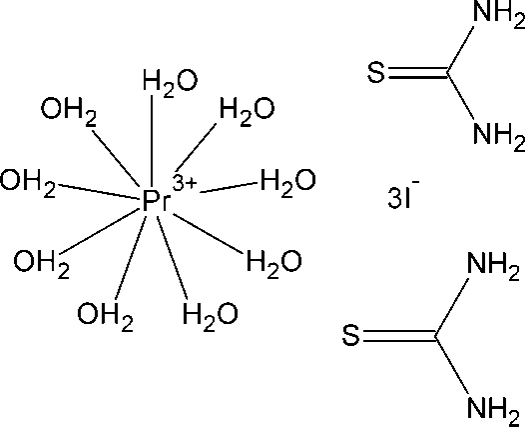


## Experimental

### 

#### Crystal data

[Pr(H_2_O)_9_]I_3_·2CH_4_N_2_S
*M* *_r_* = 836.00
Monoclinic, 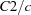

*a* = 24.934 (18) Å
*b* = 8.439 (3) Å
*c* = 14.143 (8) Å
β = 124.68 (5)°
*V* = 2447 (3) Å^3^
*Z* = 4
Ag *K*α radiation
λ = 0.56085 Å
μ = 3.16 mm^−1^
*T* = 295 K
0.20 × 0.20 × 0.20 mm

#### Data collection

Enraf–Nonius CAD-4 diffractometer
Absorption correction: ψ scan (North *et al.*, 1968 ▶) *T*_min_ = 0.405, *T*_max_ = 0.592
2309 measured reflections
2309 independent reflections
1827 reflections with *I* > 2σ(*I*)
1 standard reflection every 120 min intensity decay: 2%

#### Refinement

*R*[*F* ^2^ > 2σ(*F* ^2^)] = 0.040
*wR*(*F* ^2^) = 0.100
*S* = 0.99
2309 reflections
98 parameters
H-atom parameters constrained
Δρ_max_ = 1.24 e Å^−3^
Δρ_min_ = −0.88 e Å^−3^

### 

Data collection: *CAD-4 EXPRESS* (Enraf–Nonius, 1989 ▶); cell refinement: *CAD-4 EXPRESS*; data reduction: *XCAD4* (Harms & Wocadlo, 1995 ▶); program(s) used to solve structure: *SHELXS97* (Sheldrick, 2008 ▶); program(s) used to refine structure: *SHELXL97* (Sheldrick, 2008 ▶); molecular graphics: *Mercury* (Macrae *et al.*, 2006 ▶); software used to prepare material for publication: *WinGX* (Farrugia, 1999 ▶).

## Supplementary Material

Crystal structure: contains datablock(s) global, I. DOI: 10.1107/S1600536811054663/rk2319sup1.cif

Supplementary material file. DOI: 10.1107/S1600536811054663/rk2319Isup2.mol

Structure factors: contains datablock(s) I. DOI: 10.1107/S1600536811054663/rk2319Isup3.hkl

Additional supplementary materials:  crystallographic information; 3D view; checkCIF report

## Figures and Tables

**Fig. 1.**
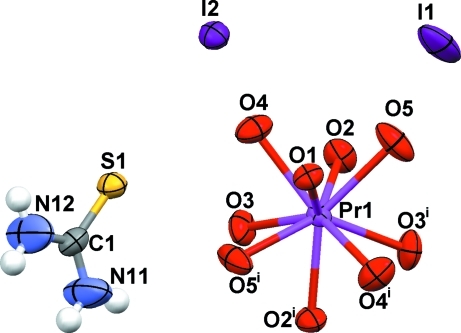
Crystal structure of I with the atom numbering scheme. Displacement ellipsoids are shown at the 50% probability level. Only independent iodide anions are shown. Symmetry code: (i) 1-x, y, 1/2-z.

**Fig. 2.**
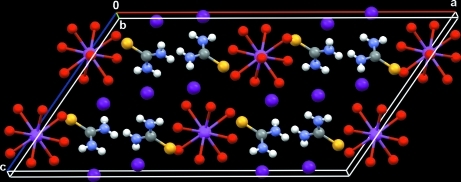
Crystal packing of I, a view along the a axis.

## supplementary crystallographic information

### Comment

The structure investigation of the interaction products of metal salts with thiourea CS(NH_2_)_2_ is very promising, because it allows to predict the possible ways of thermal decomposition of these compounds yielding oxide, sulfide and oxosulfide derivatives (Suponitsky *et al.*, 1988). The lanthanide derivatives are very promising objects of research in this regard, since the corresponding sulfides and oxosulfides are used as the activators of materials with the luminescent properties. To the present time the structure of only some thiourea derivatives of lanthanide salts has been studied in details. Systematic investigation of the previously synthesized thiourea derivatives allowed us to conclude that there are two isostructural series of lanthanide acetates (La–Pr and Nd–Lu) (Romanenko *et al.*, 1980; Romanenko *et al.*, 1981*a*; Romanenko *et al.*, 1986), and three isostructural series of lanthanide propionates (La–Pr, Nd–Tm, Yb–Lu) (Romanenko *et al.*, 1981*b*; Romanenko *et al.*, 1985). It was established the existence of complex cations in the structures, involving coordinated water molecules as well as bidentate and bridging acetate or propionate ions. It was noted that thiourea is not included into the internal sphere of complexes. The information about the synthesis and structure of thiourea derivatives of lanthanide halides is much smaller. We have obtained the compounds of thiourea with *Ln*I_3_ (*Ln* = Eu, Ho, Er) at room temperature (Antonenko *et al.*, 2011). X–ray data have been demonstrated that in the solid state these compounds are composed from [*Ln*(H_2_O)_9_]^3+^ cations (polyhedron: monocapped tetragonal antiprism), non–coordinated thiourea molecules and iodide–ions.

Herein we report the structure of thiourea adduct of nonaaquapraseodymium triiodide I (Fig. 1). In the solid state I is composed from [Pr(H_2_O)_9_]^3+^ cations (polyhedron: monocapped tetragonal antiprism), thiourea molecules and iodide anions. All mentioned species are evidently connected with H–bonds but in the presence of heavy atoms water H atoms have not been located and thus can not be discussed. The complex cation and one of the two independent iodide anions are located on a twofold axis. There is no coordination of thiourea by the lanthanide atom as through the atom S, and through the atom N as well as in the cases of compounds which have been obtained previously (Antonenko *et al.*, 2011). A packing diagram is shown in Fig. 2.

### Experimental

The synthesis of title compound was carried out at room temperature by mixing PrI_3_×9H_2_O and CS(NH_2_)_2_ at a molar ratio 1:1.7. Few drops of water were added to the reaction mixture to the formation of clear solution. After 30 days the light green crystals were identified from it. These crystals are hygroscopic; they are decomposed by water with the release of the initial thiourea. The crystals of title compound suitable for X–ray analysis were dried over the alkali in the desiccator. By complexometric titration with 0.1 *M Edta* and reverse iodometric titration with 0.1 *M* Na_2_S_2_O_3_ (Patrovsky, 1959; Kolthoff & Belcher, 1957) we established that the molar ratio of nonaaquapraseodymium triiodide and thiourea in this compound is 1:2.

### Refinement

In the presence of heavy atoms water H atoms could not be located. The hydrogen atoms bound to N atoms were placed in calculated positions with N—H = 0.86Å and refined as riding with *U*_iso_(H) = 1.2*U*_eq_(N).

### Crystal data

**Table:**

[Pr(H_2_O)_9_]I_3_·2CH_4_N_2_S	*F*(000) = 1552
*M**_r_* = 836.00	*D*_x_ = 2.269 Mg m^−^^3^
Monoclinic, *C*2/*c*	Ag *K*α radiation, λ = 0.56085 Å
Hall symbol: -C 2yc	Cell parameters from 25 reflections
*a* = 24.934 (18) Å	θ = 12–13°
*b* = 8.439 (3) Å	µ = 3.16 mm^−^^1^
*c* = 14.143 (8) Å	*T* = 295 K
β = 124.68 (5)°	Prism, light green
*V* = 2447 (3) Å^3^	0.20 × 0.20 × 0.20 mm
*Z* = 4	

### Data collection

**Table:**

Enraf–Nonius CAD-4 diffractometer	1827 reflections with *I* > 2σ(*I*)
Radiation source: fine–focus sealed tube	*R*_int_ = 0.000
graphite	θ_max_ = 20.0°, θ_min_ = 1.6°
non–profiled ω scans	*h* = −30→24
Absorption correction: ψ scan (North *et al.*, 1968)	*k* = 0→10
*T*_min_ = 0.405, *T*_max_ = 0.592	*l* = 0→17
2309 measured reflections	1 standard reflections every 120 min
2309 independent reflections	intensity decay: 2%

### Refinement

**Table:**

Refinement on *F*^2^	Primary atom site location: structure-invariant direct methods
Least-squares matrix: full	Secondary atom site location: difference Fourier map
*R*[*F*^2^ > 2σ(*F*^2^)] = 0.040	Hydrogen site location: inferred from neighbouring sites
*wR*(*F*^2^) = 0.100	H-atom parameters constrained
*S* = 0.99	*w* = 1/[σ^2^(*F*_o_^2^) + (0.0486*P*)^2^ + 1.3974*P*] where *P* = (*F*_o_^2^ + 2*F*_c_^2^)/3
2309 reflections	(Δ/σ)_max_ < 0.001
98 parameters	Δρ_max_ = 1.24 e Å^−^^3^
0 restraints	Δρ_min_ = −0.88 e Å^−^^3^

### Special details

**Table:**

Experimental. North *et al.*, 1968. Number of ψ–scan sets used was 5. Theta correction was applied. Averaged transmission function was used. Fourier smoothing - Window value 3.
Geometry. All s.u.'s (except the s.u. in the dihedral angle between two l.s. planes) are estimated using the full covariance matrix. The cell s.u.'s are taken into account individually in the estimation of s.u.'s in distances, angles and torsion angles; correlations between s.u.'s in cell parameters are only used when they are defined by crystal symmetry. An approximate (isotropic) treatment of cell s.u.'s is used for estimating s.u.'s involving l.s. planes.
Refinement. Refinement of *F*^2^ against ALL reflections. The weighted *R*–factor *wR* and goodness of fit *S* are based on *F*^2^, conventional *R*–factors *R* are based on *F*, with *F* set to zero for negative *F*^2^. The threshold expression of *F*^2^ > σ(*F*^2^) is used only for calculating *R*–factors(gt) *etc*. and is not relevant to the choice of reflections for refinement. *R*–factors based on *F*^2^ are statistically about twice as large as those based on *F*, and *R*–factors based on ALL data will be even larger.

### Fractional atomic coordinates and isotropic or equivalent isotropic displacement parameters (Å^2^)

**Table:**

	*x*	*y*	*z*	*U*_iso_*/*U*_eq_	
Pr1	0.5000	0.80167 (6)	0.2500	0.03210 (15)	
O1	0.5000	1.1003 (7)	0.2500	0.0419 (15)	
O2	0.5919 (2)	0.6295 (6)	0.2854 (4)	0.0547 (13)	
O3	0.5369 (3)	0.6201 (7)	0.4163 (4)	0.0619 (14)	
O4	0.5386 (3)	0.9090 (6)	0.1328 (5)	0.0604 (14)	
O5	0.6059 (2)	0.9151 (7)	0.4141 (5)	0.0705 (17)	
I1	0.2500	0.7500	0.0000	0.0930 (4)	
I2	0.36775 (2)	0.71928 (6)	0.45157 (4)	0.04941 (17)	
S1	0.57937 (10)	0.7171 (2)	0.66733 (17)	0.0466 (4)	
C1	0.6621 (4)	0.7453 (8)	0.7467 (7)	0.0489 (18)	
N11	0.6976 (4)	0.6708 (17)	0.7270 (10)	0.160 (6)	
H11A	0.7387	0.6912	0.7657	0.192*	
H11B	0.6812	0.5988	0.6747	0.192*	
N12	0.6906 (4)	0.8508 (17)	0.8263 (9)	0.170 (6)	
H12A	0.7319	0.8661	0.8622	0.204*	
H12B	0.6683	0.9061	0.8437	0.204*	

### Atomic displacement parameters (Å^2^)

**Table:**

	*U*^11^	*U*^22^	*U*^33^	*U*^12^	*U*^13^	*U*^23^
Pr1	0.0272 (2)	0.0302 (2)	0.0343 (3)	0.000	0.0148 (2)	0.000
O1	0.051 (4)	0.031 (3)	0.052 (4)	0.000	0.034 (3)	0.000
O2	0.050 (3)	0.051 (3)	0.058 (3)	0.013 (2)	0.028 (3)	−0.010 (3)
O3	0.075 (4)	0.058 (3)	0.040 (3)	0.021 (3)	0.026 (3)	0.019 (3)
O4	0.081 (4)	0.046 (3)	0.079 (4)	−0.017 (3)	0.060 (3)	−0.010 (3)
O5	0.039 (3)	0.058 (3)	0.075 (4)	0.006 (3)	0.009 (3)	−0.031 (3)
I1	0.0320 (4)	0.1182 (9)	0.0991 (8)	−0.0120 (4)	0.0196 (5)	0.0195 (6)
I2	0.0496 (3)	0.0431 (3)	0.0541 (3)	0.0015 (2)	0.0286 (3)	−0.0034 (2)
S1	0.0478 (10)	0.0444 (10)	0.0513 (10)	0.0068 (8)	0.0304 (9)	0.0000 (8)
C1	0.060 (5)	0.048 (4)	0.052 (4)	0.002 (4)	0.040 (4)	−0.004 (3)
N11	0.053 (5)	0.244 (15)	0.160 (10)	−0.019 (8)	0.047 (6)	−0.129 (11)
N12	0.075 (6)	0.240 (15)	0.156 (10)	−0.030 (8)	0.042 (7)	−0.151 (11)

### Geometric parameters (Å, °)

**Table:**

Pr1—O3^i^	2.503 (5)	Pr1—O1	2.520 (6)
Pr1—O3	2.503 (5)	S1—C1	1.713 (10)
Pr1—O5	2.511 (5)	C1—N11	1.240 (12)
Pr1—O5^i^	2.511 (5)	C1—N12	1.287 (11)
Pr1—O2^i^	2.512 (5)	N11—H11A	0.8600
Pr1—O2	2.512 (5)	N11—H11B	0.8600
Pr1—O4	2.512 (5)	N12—H12A	0.8600
Pr1—O4^i^	2.512 (5)	N12—H12B	0.8600
			
O3^i^—Pr1—O3	104.5 (3)	O5—Pr1—O4^i^	81.32 (19)
O3^i^—Pr1—O5	137.14 (18)	O5^i^—Pr1—O4^i^	82.9 (2)
O3—Pr1—O5	74.6 (2)	O2^i^—Pr1—O4^i^	72.02 (19)
O3^i^—Pr1—O5^i^	74.6 (2)	O2—Pr1—O4^i^	136.54 (17)
O3—Pr1—O5^i^	137.14 (18)	O4—Pr1—O4^i^	137.7 (2)
O5—Pr1—O5^i^	135.2 (3)	O3^i^—Pr1—O1	127.76 (13)
O3^i^—Pr1—O2^i^	69.67 (18)	O3—Pr1—O1	127.76 (13)
O3—Pr1—O2^i^	68.84 (18)	O5—Pr1—O1	67.59 (13)
O5—Pr1—O2^i^	139.9 (2)	O5^i^—Pr1—O1	67.59 (13)
O5^i^—Pr1—O2^i^	71.06 (17)	O2^i^—Pr1—O1	125.34 (13)
O3^i^—Pr1—O2	68.84 (18)	O2—Pr1—O1	125.34 (13)
O3—Pr1—O2	69.67 (18)	O4—Pr1—O1	68.86 (12)
O5—Pr1—O2	71.06 (17)	O4^i^—Pr1—O1	68.86 (12)
O5^i^—Pr1—O2	139.9 (2)	N11—C1—N12	115.9 (9)
O2^i^—Pr1—O2	109.3 (3)	N11—C1—S1	122.0 (7)
O3^i^—Pr1—O4	71.02 (17)	N12—C1—S1	122.0 (7)
O3—Pr1—O4	140.07 (18)	C1—N11—H11A	120.0
O5—Pr1—O4	82.9 (2)	C1—N11—H11B	120.0
O5^i^—Pr1—O4	81.32 (19)	H11A—N11—H11B	120.0
O2^i^—Pr1—O4	136.54 (17)	C1—N12—H12A	120.0
O2—Pr1—O4	72.02 (19)	C1—N12—H12B	120.0
O3^i^—Pr1—O4^i^	140.07 (18)	H12A—N12—H12B	120.0
O3—Pr1—O4^i^	71.02 (17)		

Symmetry codes: (i) −*x*+1, *y*, −*z*+1/2.
